# Nitrous Oxide Gas

**Published:** 1870-05

**Authors:** 


### NITROUS OXIDE GAS.

I have long been impressed with the idea that the reckless use of nitrous oxide would result, sooner or later, in injury to many. The facts demonstrate that my impression is correct, as the following will clearly show. I do not doubt that this agent, in competent hands, may be regarded safe, but as all know full well our profession of late, especially since the advent of rubber, has been cursed with a large amount of ignorance, that many have commenced practice without even understanding the first principles of Dental science. It is further well known, that this class of Dentists make greater pretentions than the really meritorious, and that they all, probably, with few exceptions give gas, and advertise largely. Our city is blessed with quite a number of this class of men. They make many promises, talk largely, and you would think to hear them that few, if any except themselves, knew enough to practice Dentistry. Then is it to be wondered at, that some persons should be injured, and even die at such persons hands ? The greatest wonder is that more have not died from the effects of nitrous oxide gas.

Persons are complaining to me almost daily that they went to Dr. so-and-so, and took gas and had one or more "teeth pulled," and that they had been sick afterwards, etc. I have little sympathy for some of these, for they ought to know better; but many do not, and go because some one said that they had teeth pulled and didn't know it. My observation is that fewer persons are taking it now than were a few years ago. The amount of complaint coming up against it from many is deterring others from its use.

I desire to call attention to a few cases to show that somebody is at fault, or else the gas is not all that it is thought to be. I will give only a few cases, some of which were related by a neighbor practitioner:

CASE 1.—Miss. B—— took gas. Everything seemed to work finely. She recovered soon without any difficulty, but on the next day was affected with drowsiness, so much so that it required an effort to keep her awake. This condition lasted for some time. She is very absent minded since its administration.

CASE 2.—Mrs. Mc—— had taken gas several times—the last time 8 or 9 months ago. She has, to a great extent lost her memory since, and attributes it to the gas. She was in perfect health prior to taking the gas.

CASE 3.—Mrs. B——took gas and was affected same as case 2. Has not been so long affected, but hopes for her recovery are not good.

CASE 4.—Mrs. —— took gas for the extraction of several teeth. Was affected at the time as others seemed to be. Went home (in the country) lived until the next day and died very suddenly. As to this case, no one of the profession here know anything. She died, but of what cause we do not know. The friends say "the gas killed her."

CASE 5.—Dr. W——, city, took gas and had dizziness of head for six months afterwards. Could not be induced to take it again.

CASE 6.—Miss D—— went into spasms soon after taking gas, which lasted for over sixteen hours. We have no means of knowing the result as she was taken home some distance in the country.

CASE 7.—A lady in the country took gas and could not be aroused. She lingered in this way for a few days, and died.

One more case and I am done:

A lady of this city took gas to have six or seven teeth extracted. She was of such nervous temperament that one tooth was all that could be extracted at one administration of the gas, so that it was given for each tooth. After the removal of the teeth she complained of great soreness of the throat. Swelling supervened which was very great. This lasted for several days and as it went down she began to experience trouble in the abdomen, and regions of the womb, which increased in severity until death terminated her sufferings. She lived from Saturday to Monday one week following. The physician (homœopathic) who attended her says she was not injured with the gas. What did it then?

I took a lady to a friend who gave her gas, and extracted four teeth. She went home feeling reasonably well, but took sick in a few hours which lasted two weeks.

Many other cases might be added, but it is not necessary. That many have suffered immediately after taking what those giving it call nitrous oxide gas, I have no doubt.

I never have given it. I do not assert that the gas has killed any one, but the facts as stated above show something to be wrong. Now what is it? The friend who furnished me with a portion of these cases has become alarmed, and could not be induced to give it again. If hundreds of others would follow his noble example humanity would be the gainer.

LOUISVILLE, KY. S.

